# New guidance to seekers of autism biomarkers: an update from studies of identical twins

**DOI:** 10.1186/s13229-021-00434-w

**Published:** 2021-04-19

**Authors:** John N. Constantino

**Affiliations:** Department of Psychiatry, Washington University in St. Louis, 660 S Euclid Ave, Campus Box 8504, St. Louis, MO 63110 USA

## Abstract

**Background:**

The autism spectrum disorders (ASD) are common neuropsychiatric conditions of childhood for which the vast proportion of population risk is attributable to inheritance, and for which there exist few if any replicated biomarkers.

**Main body:**

This commentary summarizes a set of recent studies involving identical (monozygotic, MZ) twins which, taken together, have significant implications for the search for biomarkers of inherited susceptibility to autism. A first is that variation-in-severity of the condition (above the threshold for clinical diagnosis) appears more strongly influenced by stochastic/non-shared environmental influences than by heredity. Second is that there exist disparate early behavioral predictors of the familial recurrence of autism, which are themselves strongly genetically influenced but largely independent from one another. The nature of these postnatal predictors is that they are trait-like, continuously distributed in the general population, and largely independent from variation in general cognition, thereby reflecting a developmental substructure for familial autism. A corollary of these findings is that autism may arise as a developmental *consequence* of an allostatic load of earlier-occurring liabilities, indexed by early behavioral endophenotypes, in varying permutations and combinations. The clinical threshold can be viewed as a “tipping point” at which stochastic influences and/or other non-shared environmental influences assert much stronger influence on variation-in-severity (a) than do the genetic factors which contributed to the condition in the first place, and (b) than is observed in typical development.

**Conclusion:**

Biomarkers identified on the basis of association with clinical symptom severity in ASD may reflect *effects* rather than *causes* of autism. The search for biomarkers of pathogenesis may benefit from a greater focus on traits that predict autism recurrence, among both clinical and general populations. In case–control studies, salient developmental liabilities should be systematically measured in both cases and controls, to avoid the erosion in statistical power (i.e., to detect differences) that can occur if control subjects carry sub-clinical aggregations of the same unmeasured traits that exert causal influences on the development of autism.

## Main text

Although the vast proportion of population risk for autism is attributable to the effects of genetic variation, the discovery of biomarkers to capture the specific biological effects of genes or to enable the identification of pre-diagnostic signatures of convergent mechanisms-of-causation across disparate pathways to the condition have lagged behind progress in molecular genetics. The association of numerous rare, de novo (germline) chromosomal and sequence variants with autism has dominated much of the landscape of autism research. Although rare, monogenic pathways to autism can serve as highly informative models of the biological effects of single genes, they do not necessarily specify convergent mechanisms relevant to the *majority* of affected individuals, i.e., those whose conditions are a result of additive (polygenic) risk inherited in a family. An important distinction between the common forms of autism that are inherited and the rare forms that rise by de novo single gene mutations is that the latter cases are almost always accompanied by intellectual disability [1], a comorbidity which characterizes only a minority of cases of autism in the population. Other important differences may include contrasts in the timing, complexity, and potential “rescue-ability” of the condition. The purpose of this communication is to alert scientists to new insights on what precisely is inherited in autism, generated in part by studies over the past several years involving identical (monozygotic) twins, which have significant implications for understanding causation in familial autism and, by extension, the ongoing search for neurolobiologic and genetic biomarkers, including polygenic risk scores.

The monozygotic (MZ) twin concordance rate for autism, on the order of 0.90, is a foundational anchor of scientific knowledge about the genetic structure of ASD. Recently, in a re-analysis of data acquired from 366 pairs of MZ twins uniformly phenotyped by standardized ratings and encompassing the full range of variation in autistic trait burden observed population-wide [2], we replicated a surprising observation [3] that despite the very high heritability of the condition itself, variation-in-severity between members of clinically affected MZ pairs was only modestly correlated, and more predominantly a function of *non-shared environmental influence*. In genetic epidemiology, non-shared (also referred to as “unique”) environmental influence is one of three principal categories that comprise the totality of causal influence on a trait or condition: (1) genetic influence (“nature”); (2) “common” or “shared” environmental influence (all social, environmental, intrauterine, and rearing factors that operate to make two members of a family alike in relation to the trait or condition); and (3) non-shared environmental influence, which operate to make two members of a family *different* in relation to the trait or condition of interest. Examples of non-shared environmental influence include (a) influential exposures experienced by one twin but not another (head trauma, infection, a particularly influential school teacher)—any of which might induce contrasting epigenetic modifications of the twins—(b) measurement error, and (c) stochastic influences, randomly determined influences on development whose main effects may be minimized by mechanisms that ensure highly evolved, expectant trajectories of growth and development, or amplified when those mechanisms are compromised.

**Fig. 1:**
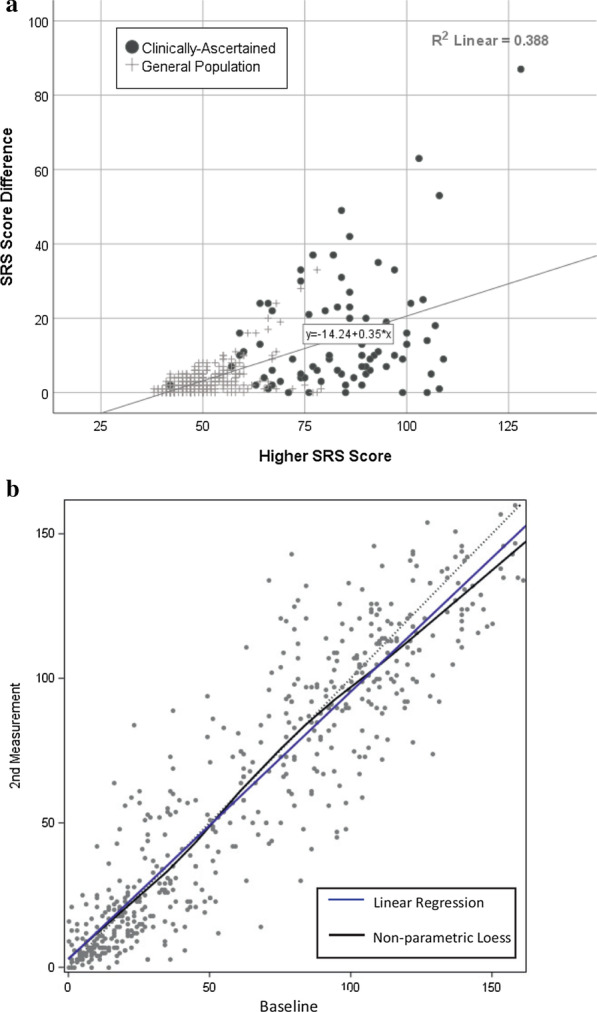
Panel **a**: Reprinted from Supplementary Materials of Castelbaum et al. *Behav Genet* 2020 [2]. Scatter plot of the SRS score of the higher-scoring member of each MZ twin pair vs. the SRS score *difference* between the MZ twins in each pair. Panel **b**: Reprinted from Wagner et al., *Child Dev* 2019 [4]. Serial maternal-report measurements of 527 children rated by the Social Responsiveness Scale 1–10 years between measurements, beginning at an average age of 9.4 years. The sample was representative of the full range of variation in autistic traits from minimal to severe, as indicated by baseline scores

The low correlations in symptom severity that we recently observed between members of clinically affected MZ twin pairs [intraclass coefficients on the order of 0.25 for total autism symptom severity; 0.24 for social communication and interaction (SCI), 0.45 for restricted interests and repetitive behavior (RRB)] contrasted with the correlations in unaffected (general population) pairs which were uniformly high (on the order of 0.75 for total severity, 0.75 for SCI and 0.69 for RRB) [2]. Our findings were in strong agreement with analyses of twin and family data derived from parents’ retrospective histories of ASD symptoms of over 1200 clinically affected children in the Autism Genetic Resource Exchange reported over a decade ago [3]. MZ twin *dissociation in severity,* which was observed exclusively in the clinically affected pairs, was unrelated to age, suggesting that the non-shared environmental influences operate early and have enduring effects on development and behavior. A plot of monozygotic (MZ) twin-twin *differences* as a function of autistic trait severity of the higher-burden twin (Fig. 1, Panel a) depicts the key observation: as the threshold for clinical-level severity is approached (approximated here by a score of 75 on the Social Responsiveness Scale, which represents an arbitrary cutoff), the MZ twin-twin contrasts are progressively (continuously) amplified to a point where randomness overtakes identical co-twin prediction (genes) as the dominant influence. It is notable that such erosion in MZ twin similarity is not observed, by way of example, for supranormal  intelligence, when examined among identical twins [4]. Furthermore, Figure 1 Panel b clarifies that what is observed in autism is not an effect of reduction in the precision of measurement of the phenotype at higher levels of severity; specification *within an affected individual* exhibits strong test–retest reliability and a high degree of long-term stability from early childhood through adulthood [5]. The observed twin-twin differences were documented by both parent-report of symptoms on a standardized quantitative trait measure, and (in a sub-set of the clinical cases) direct clinician observation using the Autism Diagnostic Observation Schedule. Although it is possible that rater contrast effects of parents contributed to some of the observed twin-twin variance among the clinical pairs, this has rarely been observed in epidemiologic samples, and the authors reported close correspondence between parent-report and direct clinician observation for even the larger twin-twin discrepancies reported by parents in the sample [2]. Despite the discrepancies in the severity of ASD in identical affected co-twins, probandwise concordance for the condition itself was well preserved (0.96) throughout the accumulated sample [2].

The major implication of a reduction in MZ trait correlation above versus below the threshold for clinical affectation is that as the causal conditions for clinical-level affectation are approached and exceeded, brain and behavioral development may become increasingly sensitive to random/stochastic perturbations that are otherwise buffered in typical development. The consequence for biomarker discovery is that if correlation with symptom severity is used as a standard for biomarker association, the association may reflect something different than what principally causes autism (i.e., genetic influence), relating instead to epiphenomena *after* the condition has been engendered by its genetic cause(s), or in other words indexing *effects* rather than *causes* of autism.

What then should be understood about the preservation of high MZ trait correlations *below* the threshold for autism, especially when it has been well established that the additive genetic causes of clinical-level impairment and sub-clinical traits largely overlap [6]? Here, in the general population, sub-clinical aggregation of autistic traits is not associated with vulnerability to stochastic (random) influences or non-shared environmental effects, and therefore variation that is captured by phenotypic measurement is a more direct index of genetic causation. Among clinical cases, vulnerability to these effects represents a signature of both impairment and increased variance, which may extend to many neuropsychiatric disorders for which genetic epidemiologic studies have historically documented substantial but largely unexplained non-shared environmental influences. The biomarker conundrum for autism (reminiscent of the Heisenberg Uncertainty Principle) is this: when the additive genetic determinants of autism accumulate to the level of crossing the clinical threshold, the ability to trace their effects on the condition may be substantially blurred. Two distinct ways of obviating this problem are to study the contributors to autism liability (1) in the general population and/or (2) before autism develops.

For inherited forms of autism (the most common kind), significant clues to the direct impact of inherited genetic risk have been derived from studies of the prediction of autism *recurrence* within families, followed by examination of those predictors among MZ twins in the general population [7]. What are the most robust predictors of autism recurrence in families? The accumulated scientific literature has identified a relatively short list which *collectively* can account for a majority share of the variation in autism recurrence risk: sub-clinical autistic traits of parents, male sex, social visual disengagement in infancy as measured by eye tracking, attention deficit hyperactivity disorder traits, and motor coordination abnormalities—these have been reviewed in detail elsewhere [7, 8]. Without exception, the monozygotic twin correlation for these recurrence predictors in the general population has been found to be extremely high ranging from 0.80 to 1.00, in keeping with the magnitude of the probandwise MZ concordance rate for a categorical diagnosis of autism. Such correlation is particularly pronounced for variation in social visual engagement, in which the eye movements of identical co-twins during the viewing of dynamic social scenes were coordinated on a tens-of-milliseconds time scale as a function of genetic similarity [9]. Remarkably, it has also been learned that the above behavioral predictors of familial recurrence—when studied together in a genetically informative context—appear largely *independent* from one another in infancy and early childhood [10], during the developmental period that precedes the median age of diagnosis of children with autism.

This set of findings supports an emerging model of biological causation that features a distinct developmental substructure for familial autism [8], and suggests that autism can arise when extremes of widely distributed inherited traits occur in *combinations* that adversely affect early social development, presumably after the time of birth and in interaction with sex and the early environment. This combinatorial landscape has not yet been specified in the National Institute of Mental Health RDoC matrix, but understanding the relationship between these and other pleiotropic influences on brain and behavioral development are a goal of future research. The postnatal interactional mechanisms suggested by this proposed developmental sub-structure for familial (polygenic) autism imply opportunity for developmental intervention between the time of birth and onset of symptoms. This notably contrasts with inferences from research on the effects of highly pathogenic single-gene mutations for which the timing of developmental gene expression places autism-causing disruptions in brain development in early fetal life (rather than postnatally)—again, however, these syndromes are typically accompanied by intellectual disability, and often with structural brain abnormality, neither of which typify familial autism.

The combinatorial model of causation described above would also account for many aspects of the observed “heterogeneity” of autism, i.e., as a convergent result of disparate permutations and combinations of inherited liability. This can be likened to the known pathophysiology of hypertension. The singular quantitative trait of variation in blood pressure can be decomposed into a finite set of underlying processes (e.g., vascular resistance, stroke volume of the heart, fluid and electrolyte balance)—each with its own genetic and mechanistic structure—for which different combinations of disruptions can produce the end-result of elevation to the clinical threshold of hypertension. Targeting disruption in cardiovascular dynamics from this granular perspective has identified numerous “levers” for the therapeutic adjustment of blood pressure, and for the identification of biomarkers which relate more closely to the contributing causes than to the presence versus absence of hypertension across all cases. An important inference of this model is that the underlying traits which contribute to autism represent extremes of evolutionarily conserved, species-adaptive behavioral variation, and therefore might be appropriately re-framed as strengths that can incur “costs” in human social adaptation when occurring in particular combinations or against specific family genetic backgrounds.

In autism, the existence of a developmental substructure as described above would imply three distinct obstacles to biomarker discovery in case–control studies: (i) heterogeneity of pathways of causation; (ii) aggregation of sub-clinical traits among controls; and (iii)confounding by the epiphenomena that produce variation in severity of the phenotype above the clinical threshold. Notably the model fits with a profound observation made decades ago among families affected by multiple-incidence autism (but never heretofore fully explained) that it was the *condition* of autism—but not the symptom profile—that bred true: different affected individuals within a family were found to vary in the profile of *relative* severity of their social, communicative, and repetitive symptoms, even though the condition itself was recurring [11]. The conclusion is that autism is genetically “fractionable,” but before it develops, not afterwards, and that genes relate more closely to developmental liabilities, not symptoms of the disorder. Combinations of developmental liabilities converge on a syndrome whose symptoms are generally strongly intercorrelated, not only when the clinical threshold is crossed but in its wide range of sub-clinical manifestations population-wide [12]. Needless to say, linking circuit-based biomarkers not only to these developmental liabilities, but to the parsimonious, continuously distributed latent trait upon which the disparate causal pathways of familial autism converge, represent very high priorities for behavioral neuroscience.

There is no assurance that linking biomarkers to the contributing developmental liabilities will be simpler or more straightforward than linkage to the convergent syndrome or its secondary manifestations. Prior research on neuropsychiatric *endophenotypes* has identified sobering limitations on the extent to which such discovery efforts accelerate understanding of causal pathways from genes to brain to behavior [13], but it is very early in the exploration of such a deconstruction of autism. Mapping and quantifying contributing causal factors has been conducted to great advantage for both hypertension and Alzheimer’s Disease, and the latter exemplifies the potential for improvement in statistical power when intermediate phenotypes (CSF biomarkers) rather than categorical states of affectation or biomarkers of the *effects* of a condition (such as cortical atrophy) are used to elucidate its causes. Moreover, behavioral comorbidities traditionally dismissed as “contaminants” of the measurement of autism are only recently being taken more seriously. Now, some of the comorbidities (eg. ADHD and Developmental Coordination Disorder) are being pursued as reflections of non-specific developmental contributions to the actual *cause* of autism, and are being incorporated meaningfully into genetic and neurobiologic research. Their timing in development, and their patterns of trait aggregation—both in the general population and in families affected by autism—provide distinct clues to whether any given "comorbidity" represents a cause, correlate, or epiphenomenon of ASD [14].

Thus, successful biomarker discovery may depend upon a reconceptualization of autism as a convergent “alternate” pathway of social brain development, engendered by diverse combinations of inherited liabilities, and characterized in clinical states by enhanced sensitivity to the effects of stochastic influences and/or non-shared environment. The new data from MZ twin studies summarized here lend new urgency for biomarker studies to ensure population-representativeness in case–control designs, to consider the confounding effects of unmeasured sub-clinical traits among controls, to shift toward a greater focus on contributing factors that predict within-family recurrence, and to exercise caution in the interpretation of association with *severity*. In relation to the latter, our observations regarding quantitative discordance in MZ twins warrants further study and deeper exploration of exactly when and how in development stochastic or non-shared environmental influences might have their most enduring effects. Studies of MZ twins discordant for autism are uncommon, because categorical discordance for diagnosis is uncommon in identical twins, and selection for such pairs introduces significant risks of ascertainment bias. A notable study of 20 epidemiologically ascertained MZ twins discordant for autism [15] explored effects of perinatal insults and reported that in three cases in which hypoxia was reported by parents to have affected only one of the twins, it was always the affected twin; however, for the remaining 17, both (*n* = 6) or neither (*n* = 11) were reported to have manifested a marker of hypoxia. We note that the data on MZ twin discordance depicted in Fig. 1a were drawn from large, representative samples, in which prematurity or perinatal insults generally resulted in exclusion from the analyses and/or the data collections themselves.

In conclusion, the notion that every inherited autistic syndrome represents one of many possible permutations and combinations of separate, genetically-influenced developmental components, creates new opportunity for both biomarker discovery and novel intervention. Relating biomarkers to these developmental liabilities and recognizing that the heritability of the condition diverges from the causes of its severity may enhance precision in the identification of robust biological signatures of the condition. And when studying a putative biomarker it is crucial to consider its relationship with contributing phenotypic liabilities not only in cases but in “controls,” so that the detection of true group differences is not confounded by failing to ascertain the presence of the same phenotypic liabilities in unaffected individuals.

An important reason for this communication is that MZ twin designs account for the *totality* (100 per cent) of causal influences on autism spectrum disorder, providing respective estimations of a) non-shared environmental influence and b) familial influence (the sum of genetic and environmental influences that result in twin-twin similarity, as described above), the sum of which equals 1.0. To place this in context, molecular genetic characterization currently accounts for less than 0.2 (20 per cent) of the total population variance for ASD. Taking stock of predictors of recurrence in autism, including developmental liabilities indexed by ADHD symptomatology, sensorimotor deficits, and other candidate endophenotypes would usher in a new era for both biomarker discovery and personalized approaches to treatment or prevention. In considering early intervention in this context, even minor improvements in the severity of a contributing liability could tip the balance of an allostatic load in favor of typical development, perhaps most potently if applied before (not after) the usual time of onset of signs and symptoms of autism. And even if contributing developmental liabilities prove to be secondary to more proximate (in utero) brain developmental processes, new understanding of the role of non-shared environmental influences offers additional hope for novel strategies to ameliorate the severity of the condition in affected individuals. This may include buffering the effect of stochastic influences on brain and behavioral development, which have long been implicated in psychopathology, and for which excess vulnerability may represent a signature of “clinical-level impairment” not only in autism but in other neuropsychiatric conditions.

## Data Availability

There are no restrictions on materials or any materials transfer agreements.
